# Supplementary material for: Distribution of Glycerophospholipids in the Adult Human Lens
Source: Biomolecules. 2018 Nov 22;8(4):156. doi: 10.3390/biom8040156 (PMC6315977; doi:10.3390/biom8040156)
Supplement: Supplementary file 1 [file biomolecules-08-00156-s001.pdf]

Supplementary Material

Distribution of Glycerophospholipids in the Adult Human Lens

Jo Ann Seng[1], Jessica R. Nealon[2,3] , Stephen J. Blanksby[4] and Todd W. Mitchel[2,3]*

[1] School of Chemistry, Faculty of Science, Medicine and Health, University of Wollongong, NSW 2522, Australia.

[2] School of Medicine, Faculty of Science, Medicine and Health, University of Wollongong, NSW 2522, Australia.

[3] Illawarra Health and Medical Institute, Wollongong, NSW 2522, Australia.

[4] Central Analytical Research Facility, Institute for Future Environments, Queensland University of Technology, Brisbane, QLD 4000, Australia.

* Correspondence: toddm@uow.edu.au; Tel.: +61 2 4221 5443

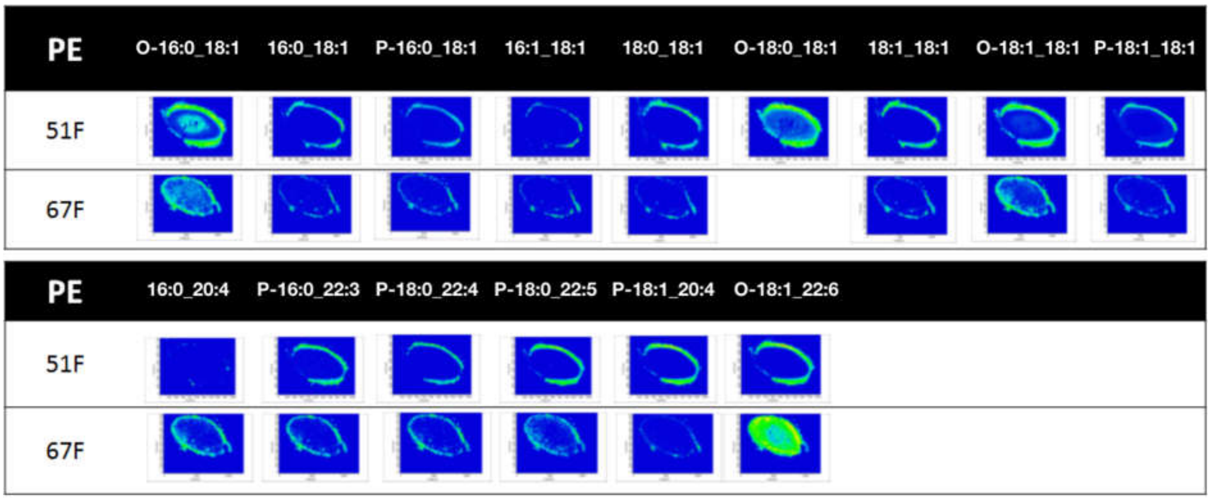

Figure S1: Distribution of all PE species in a 51- and 67-year old human lens.

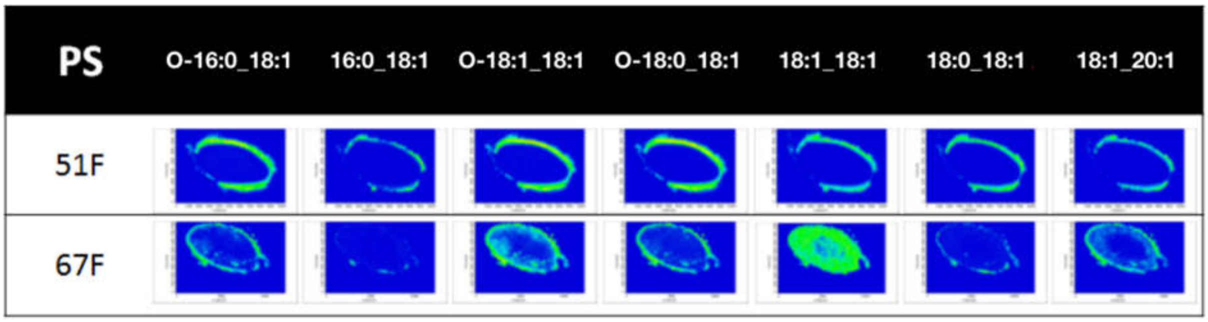

Figure S2: Distribution of all PS species in a 51- and 67-year old human lens.

Table S1: List of lipids detected in [M-H]$^-$ with *m/z* measured experimentally and expected theoretical *m/z*. The mass error is shown in ppm.

Lipid assignment	*m/z* measured	*m/z* expected	Δ m (ppm)
PE O-16:0_18:1	702.53613	702.543767	0.99998913
PE 16:0_18:1	716.5199	716.52303	0.999995632
PE P-16:0_18:1	700.56293	700.52812	1.000049691
PE 16:1_18:1	714.57379	714.50738	1.000092945
PE 18:0_18:1	744.51642	744.55433	0.999949084
PE O-18:0_18:1	730.56671	730.57507	0.999988557
PE 18:1_18:1	742.53204	742.53868	0.999991058
PE O-18:1_18:1	728.55603	728.55942	0.999995347
PE P-18:1_18:1	726.57587	726.54377	1.000044182
PE 16:0_20:4	738.57941	738.50738	1.000097535
PE P-16:0_22:3	752.55743	752.559423	0.999997352
PE P-18:0_22:4	778.5686	778.57507	0.99999169
PE P-18:0_22:5	776.56927	776.55942	1.000012684
PE P-18:1_20:4	748.54004	748.52812	1.000015925
PE O-18:1_22:6	774.56134	774.54377	1.000022684
PS O-16:0_18:1	746.53186	746.53359	0.999997683
PS 16:0_18:1	760.50745	760.51286	0.999992886
PS O-18:1_18:1	772.54498	772.54925	0.999994473
PS O-18:0_18:1	774.56134	774.56489	0.999995417
PS 18:1_18:1	786.52686	786.52851	0.999997902
PS 18:0_18:1	788.53967	788.54416	0.999994306
PS 18:1_20:1	814.52814	814.55981	0.99996112
LPE O-16:0	438.2962	438.29845	0.999994867
LPE O-18:0	466.30667	466.32975	0.999950507
LPE O-18:1	464.31149	464.31425	0.999994056
LPE P-18:1	462.30154	462.29845	1.000006684
PA O-16:0_18:1	659.49872	659.50157	0.999995679
PA O-18:0_18:1	687.53821	687.53287	1.000007767
